# Extracellular Nucleic Acids in Urine: Sources, Structure, Diagnostic Potential

**Published:** 2015

**Authors:** O. E. Bryzgunova, P. P. Laktionov

**Affiliations:** Institute of Chemical Biology and Fundamental Medicine, Siberian Branch of the Russian Academy of Sciences, 8 Lavrentiev Avenue, 630090, Novosibirsk, Russia; E.N. Meshalkin Novosibirsk Research Institute of Circulation Pathology, st. Rechkunovskaya 15, 630055, Novosibirsk, Russia

**Keywords:** urinary cell-free DNA and RNA, apoptosis, necrosis, active secretion, urine nucleases, cell-free NA based non-invasive diagnostics

## Abstract

Cell-free nucleic acids (cfNA) may reach the urine through cell necrosis or apoptosis, active secretion of nucleic acids by healthy and tumor cells of the urinary tract, and transport of circulating nucleic acids (cir- NA) from the blood into primary urine. Even though urinary DNA and RNA are fragmented, they can be used to detect marker sequences. MicroRNAs are also of interest as diagnostic probes. The stability of cfNA in the urine is determined by their structure and packaging into supramolecular complexes and by nuclease activity in the urine. This review summarizes current data on the sources of urinary cfNA, their structural features, diagnostic potential and factors affecting their stability.

## SOURCES OF URINARY CELL-FREE NUCLEIC ACIDS (FIGURE)

**Fig. 1:**
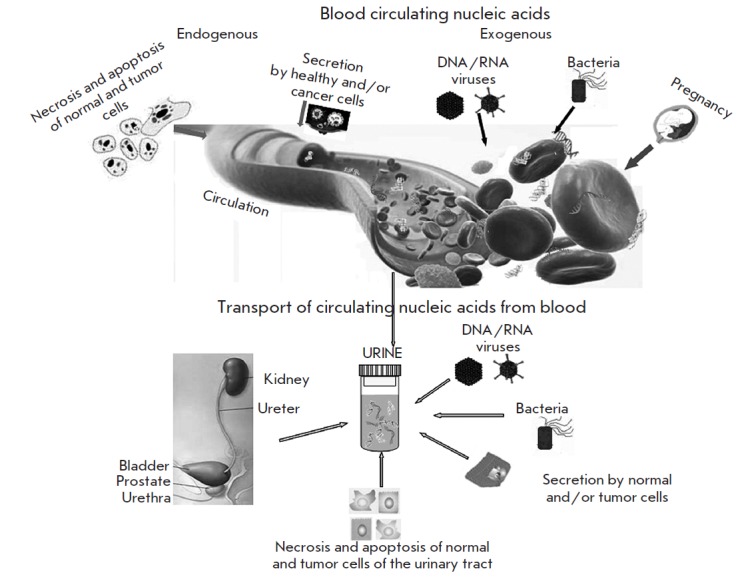
Sources of cell-free nucleic acids in the urine and blood

Cell-free nucleic acids (cfNA) may get urine as a result of renal cfNA transport from the blood or directly from the cells that came into contact with this biological fluid. The reviews [1, 2] summarize the mechanisms of generation and general properties of cell-free and circulating NA in the blood. Apoptosis is considered to be the main source of cfNA. NAs circulate in the blood as a part of complexes with biopolymers and may be packaged in membrane structures [1, 3]. Circulating DNA is highly fragmented, and the fragments’ size is proportional to a nucleosome [1]. The blood contains mRNA and ribosomal RNA, as well as non-coding RNA and miRNA, which can circulate both as part of nucleoprotein complexes and as part of membrane-coated microparticles, including exosomes [4-6]. Transport of nucleic acids from the blood into the primary urine implies the transport of the components from the afferent artery into the renal corpuscle. Glomerular filtration of plasma, which is responsible for this process, is limited by the permeability of the basal membrane and slit membranes between podocytes pedicles. For example, only complexes smaller than 6.4 nm in diameter [7] and with a molecular weight no greater than 70 kDa [8] can enter the nephron lumen; it corresponds to DNA of about 100 bp in size. The size of the pores in the glomerular barrier is about 30 A; even though shut-like pores with a 110–115 A radius have been detected, they are very rare [9]. Negatively charged molecules, such as polyanions in the basal membrane and sialoglycoproteins in the lining on podocytes surface and between their pedicles [7], play an important role in the passage of substances through the juxtaglomerular apparatus. It is known that DNA [10-12] and RNA [13-15] are primarily present in the blood as part of supramolecular complexes, such as nucleosomes [1], RNA complexes with blood lipoproteins [16, 17], or larger membrane-protected microparticles and exosomes [4] or apoptotic bodies. However, the size of a mononucleosome exceeds the size of even the largest pores of the kidney barrier and, therefore, mononucleosomes in their classical configuration are unable to pass through the barrier.

The overall health of a patient can also affect the transport of nucleic acids from the blood. An increase in the amount of DNA in the urine of patients with acute pancreatitis [18] was reported as early as in 1967, and in 2012 the urine of smokers was shown to contain more DNA than that of non-smokers (9.46 and 9.04 ng/mL for women, respectively; 4.96 and 2.93 ng/mL for men, respectively) [19]. Murine experiments have demonstrated that upon intraperitoneal administration of dying cells a portion of DNA avoids intracellular degradation and phagocytosis, circulates in the blood as a polymer, and is partially excreted with urine in acid-insoluble form [20].

A study of the degradation products of [32P]-labeled DNA of phage λ introduced into the peritoneal cavity of mice showed that the majority of these products were re-used by the cells or were hydrolyzed into acid- soluble fragments, and only ~3.2% were excreted in the urine over 3 days. A small portion of the introduced DNA (0.06%) was detected in the acid-insoluble fraction of urinary nucleic acid (length ≥15–20 bp).

It should also be stressed, however, that the excretion of “unprotected” purified DNA/RNA may be different from that of DNA/RNA from dying cells. Necrotic and especially apoptotic DNA is bound to proteins and is much better protected from nucleases than the purified DNA used in the model system. At the same time, DNA/RNA-binding proteins may have both positive and negative effects on the transportation of DNA/RNA through the kidney barrier. These assumptions are supported by the introduction of human Raji lymphoma cells whose apoptosis has been induced by γ-radiation into the abdominal cavity of mice; the urine of the experimental animals contained human Alu-sequences that were absent in the urine of the control animals (which did not receive injections of Raji cells) [20].

Another proof of circulating DNA transport from the blood into the urine is the specific Y-chromosomal DNA detected in the urine of women who have received blood from male donors [20]. Furthermore, the urine of women carrying a male fetus also contains specific Y-chromosomal DNA [20, 21]. The fetal DNA in the maternal urine was found to be considerably shorter than that in her plasma [21]. Another confirmation of polymeric DNA transport from the blood into the urine was obtained by analysis of cell-free DNA of cancer patients. It is known that 80–90% of pancreatic and intestine tumors carry mutant forms of the *K-ras *gene, which were found by Botezatu *et al*. [20] in cell-free DNA in the urine of patients with pancreatic (stage IV) and colorectal (stage III–IV) cancer. The concentration of tumor DNA in the urine was quite high; the mutant* K-ras *gene was detected in urinary cfDNA in five of the eight patients with pancreatic cancer and in four out of five patients with colorectal adenocarcinomas [20]. The feasibility of DNA transport from the blood into the urine was demonstrated in experiments which detected the presence of *Mycobacterium tuberculosis* DNA in the urine of patients with tuberculosis [22, 23].

Therefore, DNA fragments, 50–100 bp in size, which are detected in the blood are, apparently, partially protected by histones, but can, nevertheless, reach the urine. In addition, it has been suggested that the binding of DNA to histones, e.g. to H3K27me2, may contribute to the export of cell-free DNA [24].

Another obvious and, apparently, primary source of cfDNA and cfRNA in the urine is apoptosis/necrosis of urinary tract cells. Indeed, under normal circumstances up to 3×10^6^ of the bladder and urinary tract epithelium cells can be excreted into the urine within 24 hours (calculations based on the Kakhovsky–Addis method) [25].These cells and endothelial cells may partially enter apoptosis, and fragmented apoptotic DNA/RNA from the cells will inevitably reach the urine [20]: e.g., after transplantation of kidneys from male donors, the concentration of Y-chromosomal DNA in women’s urine increases in the case of rejection and returns to normal levels after the inhibition of the immune response against the transplant [26-28]. MALDI-TOF-mass spectrometry revealed the presence of SNP-alleles of the donor kidney in the urinary cell-free DNA [29]. Mutations and microsatellite disorders specific to malignant renal [30] and bladder [31-33] tumors and aberrantly methylated DNA specific to prostate [34, 35] and bladder tumors [33, 36-40] were detected in cell-free DNA in the urine of patients with urogenital cancers. The urine of patients with gynecological and urological diseases or HIV contains HPV DNA that affects deep layers of the skin and the mucous membranes of the internal organs [41]. In the case of bladder cancer, the urinary cell-free DNA contains not only genomic, but also mitochondrial DNA sequences [42].

The concentration of RNA in human urine is 20–140 ng/mL [43, 44]. Another confirmation that cfRNA appear in the urine as a result of apoptosis/necrosis of urogenital tract cells is the detection of survirin, cytokeratin 20, mucin 7, and Ki-67mRNAs in the urine of patients with bladder cancer and urinary tract infections [45, 46]. We were unable to find any data on RNA transport from the circulating blood into the urine.

Strictly speaking, the data on the presence of onco-/ fetus-specific NA in the urine do not provide a direct answer to the question of what portion of cfNA originate from the apoptosis/necrosis of the cells that line the urinary tract (it should be noted that cells of prostatic origin constitute no more than 10% of the total urine cell pool [47]). The available data on the concentration of tumor-specific NA in the urine and blood of patients with urogenital system cancers indicate that the transport of tumor-specific cfNA from the blood does not define the concentration of these cfNA in the urine. For example, methylated forms of *GSTP1* and *RASSF1A *genes are detected in 15 and 65% of the urine samples of patients with renal cancer, but only in 6 and 11% of the serum samples of the patients, respectively [48]; i.e., these marker DNAs cannot come from the blood and most likely are transported directly into the urine. These and other [49, 50] data confidently demonstrate that the major portion of cancer-specific cell-free NA in the urine of patients with urogenital tract cancers does not come from the blood and, is, apparently, the result of direct transport of tumor cells or their breakdown products into the urinary tract of diffusion through kidney tissues.

## PARTICULAR FEATURES OF URINARY NUCLEIC ACIDS STRUCTURE AND COMPOSITION

Cell-free DNA fragments in the urine can be divided into two groups based on their size: heterogeneous high-molecular-weight DNA (1 kbp or higher) and relatively homogeneous low-molecular-weight DNA (150–250 bp) [20, 43, 51, 52]. Low-molecular-weight DNAs of 10–150 and 150–200 bp were also found in the urine [53].

Only few papers are devoted to the study of DNA and RNA in the cell-free fraction of the urine, whereas the bulk of research involves the search for cancer- specific markers in total urine or in urine cells only. Tumor-specific changes in DNA identified in DNA circulating in the blood are present in urinary cfDNA as well: e.g., point mutations, microsatellite composition disorders, characteristic methylation profile of oncogenes, presence of viral DNA [30, 33, 36, 41].

DNA markers were mainly analyzed by PCR. Microsatellite adjustments (in one or more of the 28 markers (D1S251, HTPO, D3S1317, D3S587, D3S1560, D3S1289, D3S1286, D3S1038, D4S243, FGA, CSF, ACTBP2, D8S348, D8S307, D9S747, D9S242, IFNa, D9S162, D11S488, THO, vWA, D13S802, MJD, D17S695, D17S654, D18S51, MBP, D21S1245) were found in the urine of 76% of patients with kidney tumors [30]. 27% of patients with bladder tumors [31] had at least one disorder of microsatellite DNA (D4S243, D9S747, D9S171, D17S695, D17S654).

Cell-free DNA with mutations in the *FGFR3 *gene were found in the urine of 34.5% of patients with bladder cancer [33]; *P53*, in 52.9% of patients with liver cancer [54]; *K-ras*, in 95% of patients with colon cancer [55].

Aberrantly methylated DNA typical for prostate tumor cells (*GSTP1*gene) were found in the urine of 36% of prostate cancer patients [34, 56] and 3.2% of those with benign prostatic hyperplasia [35]. Changes in methylation were observed in a number of genes of the urinary cell-free DNA of bladder cancer patients:* CDKN2A *(46.7%), *ARF *(26.7%), *GSTP1 *(46.7%), *MGMT* (26.7%), *RARβ2 *(60%), *TIMP3 *(46.7%), *CDH1 *(66.7%),* RASSF1A *(53%) and *APC *(53%) [37]. Moreover, simultaneous determination of the methylation status of four genes, *MYO3A*, *CA10*, *NKX6*, *SOX11 *or *MYO3A*, *CA10*,* NKX6*, *DBC1, *in urinary cfDNA allows one to detect bladder cancer with a high sensitivity (81.3%) and specificity (97.3%), whereas simultaneous determination of the methylation status of five genes, *MYO3A*, *CA10*,* NKX6*, *DBC1*, *SOX11 *or *MYO3A,CA10*, *NKX6*, *DBC1*,* PENK, *enables the detection of bladder cancer with a sensitivity of 85.2% and a specificity of 94.5% [40].

HPV type 16 DNA was detected in the urine of women with cervical abnormalities, including 88.8% of cancer patients, 76.5% of patients with high-grade lesions, and 45.5% of patients with low-grade lesions [57]. The urine of patients with prostate cancer who underwent surgical treatment contained HPV DNA in 50% of cases [58].

In respect to marker cfRNA, quantitative RT-PCR detected specific Ki-67 mRNA, which was absent from the urine of five healthy donors, in two out of the four patients with bladder cancer and two out of the four patients with urinary tract infections [46]. Furthermore, RT-PCR detected mRNA of survirin (sensitivity 90.4%, specificity 94.7%), cytokeratin 20 (sensitivity 82.6%, specificity 97.4%), and mucin 7 (sensitivity 62.6%, specificity 94.7%) (*P* < 0.001) in the urine of bladder cancer patients. The combination of these three markers enables the detection of bladder cancer with a sensitivity of 100% at a specificity of 89.5% [45].

Determination of the concentration of CD147, BIGH3, and STMN1 mRNA in cell-free urine supernatant (after centrifugation of the total urine at 10,000 rpm) revealed that the concentration of mRNA is 2–67 times higher in patients with urothelial bladder cancer than in healthy donors [59].

AMACR (α-methylacyl coenzyme A racemase) mRNA is a promising prostate cancer-specific marker specific. The detection of AMACR mRNA in the urine sediment of 92 men, 43 of whom were diagnosed with prostate cancer, enables the identification of patients with a sensitivity of 70% and specificity of 71%, whereas the determination of PCA3 mRNA enables 72% sensitivity and 59% specificity [60]. Simultaneous determination of AMACR and PCA3 mRNA increases the sensitivity and specificity of the test to 81 and 84%, respectively.

Analysis of the ratios of ETS2 (v-ets erythroblastosis virus E26 oncogene homolog 2) mRNA and uPA (urokinase plasminogen activator) mRNA in cell-free RNA in total urine (without centrifugation/precipitation of the cells) make it possible to diagnose bladder cancer with 100% specificity and 75.4% sensitivity [61].

However, the use of urinary mRNA for the development of diagnostic systems for various diseases remains quite a challenge, since the urine contains a lot of nucleases, including RNases (their diversity is described in the next chapter). High concentration of enzymes that hydrolyze RNA complicates the processing of cell-free RNA, including the isolation stage. Unlike long mRNAs, miRNAs are more resistant to nucleases due to their small size (20–25 nucleotides), and the ability to form stable complexes with biopolymers or to be packed into different microparticles, e.g., exosomes [4]. The urine indeed contains m-, sca-, sno-, sn-, pi-, and miRNAs, including those in exosomes [4, 62]. Based on these data, more and more researchers are trying to develop test systems for the diagnosis of various cancers by analyzing miRNA in urine.

For example, it has been shown that the ratio of miRNA-126 and miRNA-152 concentrations in the urine enables detection of bladder cancer at 82% specificity and 72% sensitivity [63]. Determination of microRNA- 210, -10b, and 183 concentrations increases the specificity of the detection of bladder cancer to 91% with a sensitivity of at least 71% [64].

More than 204 group-specific miRNA were found in the urine of healthy donors, cancer patients, and pregnant women; some of them may be potential markers (e.g., miR-515-3p, 335, 892a, 509-5p, 223*, 873, 302d, 616*, 134) [44].

The level of microRNA 483-5p expression in the cellfree fraction of the urine was found to be significantly higher (Mann-Whitney, *P *= 0.013) in prostate cancer patients [65].

The study of miRNA of the epithelial-mesenchymal transition (EMT) [66] in urine sediment and supernatant from 51 bladder cancer patients and 24 healthy volunteers revealed a decrease in the amount of miRNA- 200, miRNA-192, and -155 families in the sediment, as well as decreased expression of miRNA-192 and increased expression of miRNA-155 in the urine supernatant of the patients. In addition, the level of expression of miRNA-200, miRNA-205, and miRNA-192 families in the urine sediment of the patients was significantly correlated with the expression of EMT markers in the urine, including mRNA of zinc finger E-box-binding homeobox 1, vimentin, transforming growth factor-1, and the homolog gene of Ras family (member A). The levels of miRNA-200c and miRNA- 141 in the urine sediment of the patients returned to normal after the removal of the bladder tumor.

## DNA- AND RNA-HYDROLYZING ENZYMES IN THE URINE

Human urine is a suitable environment for the functioning of NA-hydrolyzing enzymes: adult daily urine contains 2.0–4.0 g of potassium, 100–400 mg of calcium, 50–150 mg of magnesium, 3.6 g of sodium, 270–850 μg of zinc [25] and the pH value of urine normally ranges from 5.0 to 7.0.

DNase I is the major DNA-hydrolyzing enzyme in the urine [67-70], as well as in the blood [1], and its activity in the urine is more than 100-fold higher than its activity in serum [71] and amounts to 400•€1200 act. U/L (specific activity of DNase I is 2000 act. U/mg, in blood 4.4 ± 1.8 act. U/L). Cell-free DNA in the urine can be hydrolyzed by all DNase I isoforms, which are known to differ in pI value, primary structure, and/ or content of sialic acid [72]. In addition, genetic polymorphism of DNase I in urine was reported in [69]. A murine model experiment demonstrated that the concentration of DNase I in the urine can significantly increase with the onset of systemic lupus erythematosus (from 24 to 521 ng/mL), thereby indirectly reflecting the disorders occurring in the body [73]. The activity of DNase I in the blood is inhibited by actin [68, 74, 75]: however, actin concentration in the urine is, apparently, significantly lower than that in the blood (the concentration of actin is determined based on the concentration of 3-methylhistidine, a specific metabolite of actin and myosin) [76].

The urine also contains DNase II [70, 71, 77]. The activity of DNase II in human urine is *ca*. 30 -times lower than that of DNase I [77]. At the same time, it is 1.5–5 times higher than in the blood [78] and amounts to *ca*. 13–40 act. U/L of the urine.

In addition to DNases, the urine also contains phosphodiesterase I, which has a pH-optimum of 9.0 (the enzyme is stable at pH 3.0 to 11.0) [71, 79].

As for RNA-hydrolyzing enzymes of the urine, unfortunately, their studies were conducted primarily in the 20th century (1970s–90s.). RNase 2 is the most abundant RNA-hydrolyzing enzyme in human urine, where its concentration is *ca. *20 times higher than that of RNase I. The molecular weight of RNase 2 as determined by electrophoresis in SDS-PAGE and gel filtration is 32 kDa and 38, respectively: the pH-optimum is in the range of 7.2–7.6 [80].

Ribonuclease I (RNase I) is the second most abundant RNA-hydrolyzing enzyme in the urine [81]. The molecular weight of this enzyme is ~16 kDa, the enzyme is active at pH 7.0, and is inhibited by Cu^2+^, Hg^2+^ and Zn^2+^ ions. RNase I is a pyrimidine-specific enzyme, and it hydrolyzes poly(C) and poly(U) more effectively than poly(A) and poly(G). RNase I is also able to hydrolyze RNA:DNA heteroduplexes [82].

In addition to RNases 2 and 1, human urine also contains RNase C and U with pH optimum of 8.5 and 7.0, respectively [83], as well as RNase 7, UL, US, UpI-1, and UpI-2. RNase C (33 kD) is a glycoprotein, which preferably hydrolyzes synthetic poly(C) homopolymer, and is similar to mammalian pancreatic RNases. RNase U (18 kDa) is also a glycoprotein and uses RNA as a substrate, but it is almost inactive against poly(C) and has a lower homology with pancreatic RNases. In terms of amino acid composition, this enzyme is similar to human spleen RNase. Other researchers also found RNAases with a molecular mass of 33 [84] and 21.5 kDa [85], a pH optimum of 6.5, and a more efficient hydrolysis of poly(C) in human urine. RNase 7 (14.5 kDa) is present in the urine in concentrations of 235–3467.2 mg/L [86]. RNase 7 exhibits antibacterial activity at alkaline pH values.

Pyrimidine-specific RNase UL (38 kDa) and US (13 kDa), with pH optima of 8.0 and 6.75, respectively, were found in the urine of adult individuals [87]. The urine of pregnant women contained RNase UpI-1 (34 kDa) and UpI-2 (38 kDa) with pH-optima of 7.7 and 6.6, respectively [88].

Therefore, cell-free DNA and RNA are heterogeneous with respect to their size and composition. They may appear in the urine both from the blood and from the cells of the urogenital system, mainly through apoptosis, necrosis, oncosis, and active secretion (as a part of exosomes). The biological function of urinary cellfree nucleic acids has not been investigated, but DNA, RNA, and small RNA are of interest for early noninvasive diagnostics of oncological diseases of various etiologies.

## Abbreviations

cfDNA: cell-free DNA
cfNA: cell-free nucleic acids
cfRNA: cell-free RNA
NA: nucleic acids
cirNA: circulating nucleic acids

## References

[1] Bryzgunova O., Laktionov P. (2014). Supplement Series B: Biomed. Chem.. Biochemistry (Moscow)..

[2] Fleischhacker M., Schmidt B. (2007). Biochim. Biophys. Acta..

[3] van der Vaart M., Pretorius P. (2008). Ann. N.Y. Acad. Sci..

[4] Li M., Zeringer E., Barta T., Schageman J., Cheng A., Vlassov A. (2014). Phil. Trans. R. Soc. B..

[5] Sita-Lumsden A., Fletcher C., Dart D., Brooke G., Waxman J., Bevan C. (2013). Biomark. Med..

[6] Rykova E., Morozkin E., Ponomaryova A., Loseva E., Zaporozhchenko I., Cherdyntseva N., Vlassov V., Laktionov P. (2012). Expert Opin. Biol. Ther..

[7] Pokrovsky V., Korot’ko G. (1997). Human Physiology. M.: Medicine,.

[8] Lote C. (1994). Principles of Renal Physiology. London: Chapman & Hall.

[9] Tencer J., Frick I., Oquist B., Alm P., Rippe B. (1998). Kidney Int..

[10] Holdenrieder S., Stieber P., Bodenmüller H., Busch M., von Pawel J., Schalhorn A., Nagel D., Seidel D. (2001). Ann. N.Y. Acad. Sci..

[11] Kiroi K., Tanaka C., Toi M. (1999). Breast Cancer..

[12] Lin J., Fan R., Zhao Z., Cummings O., Chen S. (2013). Am. J. Surg. Pathol..

[13] Ng E., Tsui N., Lam N., Chiu R., Yu S., Wong S., Lo E., Rainer T., Johnson P., Lo Y. (2002). Clin. Chem..

[14] Halicka H., Bedner E., Darzynkiewicz Z. (2000). Exp. Cell Res..

[15] Hasselmann D., Rappl G., Tilgen W., Reinhold U. (2001). Clin. Chem..

[16] Gahan P., Stroun M. (2010). Cell Biochem. Funct..

[17] Vickers K., Palmisano B., Shoucri B., Shamburek R., Remaley A. (2011). Nat. Cell Biol..

[18] Sorenson G. (2000). Clin. Cancer Res..

[19] Simkin M., Abdalla M., El-Mogy M., Haj-Ahmad Y. (2012). Epigenomics..

[20] Botezatu I., Serdyuk O., Potapova G., Shelerov V., Alechina R., Molyaka Y., Anan’ev V., Bazin I., Garin A., Narimanov M. (2000). Clin. Chem..

[21] Koide K., Sekizawa A., Iwasaki M., Matsuoka R., Honma S., Farina A., Saito H., Okai T. (2005). Prenatal Diagnosis..

[22] Tuuminen T. (2012). Front. Immunol..

[23] Peter J., Green C., Hoelscher M., Mwaba P., Zumla A., Dheda K. (2010). Curr. Opin. Pulm. Med..

[24] Peters D., Pretorius P. (2011). Clin. Chim. Acta..

[25] Chirkin A., Okorokov A., Goncharik I. (1993). Diagnostic guide to physician. Minsk: Belarus, 1993. 688 p..

[26] Zhang J., Tong K., Li P., Chan A., Yeung C., Pang C., Wong T., Lee K., Lo D. (1999). Clin. Chem..

[27] Zhong X., Hahn D., Troeger C., Klemm A., Stein G., Thomson P., Holzgreve W., Hahn S. (2001). Ann. N.Y. Acad. Sci..

[28] Zhang Z., Ohkohchi N., Sakurada M., Mizuno Y., Miyagi T., Satomi S., Okazaki H. (2001). Transplantation Proc..

[29] Li Y., Hanh D., Wenzel W., Hanh S., Holzgreve F. (2006). Ann. N.Y. Acad. Sci..

[30] Eisenberger C., Schoenberg M., Enger C., Hortopan S., Shah S., Chow N., Marshall F., Sidransky D. (1999). J. Natl. Cancer Inst..

[31] Utting M., Werner W., Dahse R., Schubert J., Junker K. (2002). Clin. Cancer Res..

[32] Mao L., Lee D., Tockman M., Erozan Y., Askin F., Sidransky D. (1994). Proc. Natl. Acad. Sci. USA..

[33] Karnes R., Fernandez C., Shuber A. (2012). Mayo. Clin. Proc..

[34] Goessl C., Krause H., Muller M., Heicappell R., Schrader M., Sachsinger J., Miller K. (2000). Cancer Research.

[35] Jeronimo C., Usadel H., Henrique R., Silva C., Oliveira J., Lopes C., Sidransky D. (2002). Urology..

[36] Goessl C., Muller M., Straub B., Miller K. (2002). Eur. Urology..

[37] Hoque M., Begum S., Topaloglu O., Chatterjee A., Rosenbaum E., Criekinge W., Westra W., Schoenberg M., Zahurak M., Goodman S., Sidransky D. (2006). J. Nat. Cancer Inst..

[38] Reinert T., Modin C., Castano F., Lamy P., Wojdacz T., Hansen L., Wiuf C., Borre M., Dyrskjot L., Orntoft T. (2011). Clin. Cancer Res..

[39] Reinert T. (2012). Adv. Urol..

[40] Chung W., Bondaruk J., Jelinek J., Lotan Y., Liang S., Czerniak B., Issa J. (2011). Cancer Epidemiol. Biomarkers Prev..

[41] Vorsters A., Micalessi I., Bilcke J., Ieven M., Bogers J., van Damme P. (2012). Eur. J. Clin. Microbiol. Infect. Dis..

[42] Ziegler A., Zangemeister-Wittke U., Stahel R. (2002). Cancer Treatment Rev..

[43] Bryzgunova O., Skvortsova T., Kolesnikova E., Starikov A., Rykova E., Vlassov V., Laktionov P. (2006). Ann. N.Y. Acad. Sci..

[44] Weber J., Baxter D., Zhang S., Huang D., Huang K., Lee M., Galas D., Wang K. (2010). Clin. Chem..

[45] Pu X., Wang Z., Chen Y., Wang X., Wu Y., Wang H. (2008). J. Cancer Res. Clin. Oncol..

[46] Menke T., Warnecke J. (2004). Ann. N.Y. Acad. Sci..

[47] Truong M., Yang B., Jarrard D. (2013). J. Urology..

[48] Hoque M., Begum S., Topaloglu O., Jeronimo C., Mambo E., Westra W., Califano J., Sidransky D. (2004). Cancer Research.

[49] Payne S., Serth J., Schostak M., Kamradt J., Strauss A., Thelen P., Model F., Day J., Liebenberg V., Morotti A. (2009). Prostate..

[50] Goessl C., Muller M., Heicappell R., Krause H., Miller K. (2001). Ann. N.Y. Acad. Sci..

[51] Su Y., Wang M., Brenner D., Ng A., Melkonyan H., Umansky S., Syngal S., Block T. (2004). J. Mol. Diagn..

[52] Su Y., Wang M., Aiamkitsumrit B., Brenner D., Block T. (2005). Cancer Biomarkers..

[53] Melkonyan H., Feaver W., Meyer E., Scheinker V., Shekhtman E., Xin Z., Umansky S. (2008). Ann. N.Y. Acad. Sci..

[54] Lin S., Dhillon V., Jain S., Chang T., Hu C., Lin Y., Chen S., Chang K., Song W., Yu L. (2011). J. Mol. Diagn..

[55] Su Y., Wang M., Brenner D., Norton P., Block T. (2008). Ann. N.Y. Acad. Sci..

[56] Bryzgunova O., Morozkin E., Yarmoschuk S., Vlassov V., Laktionov P. (2008). Ann. N.Y. Acad. Sci..

[57] Daponte A., Pournaras S., Mademtzis I., Hadjichristodoulou C., Kostopoulou E., Maniatis A., Messinis I. (2006). J. Clin. Virol..

[58] Zambrano A., Kalantari M., Simoneau A., Jensen J., Villarreal L. (2002). Prostate..

[59] Bhagirath D., Abrol N., Khan R., Sharma M., Seth A., Sharma A. (2012). Clin. Chim. Acta..

[60] Ouyang B., Bracken B., Burke B., Chung E., Liang J., Ho S. (2009). J. Urol..

[61] Hanke M., Kausch I., Dahmen G., Jocham D., Warnecke J. (2007). Clin. Chem..

[62] Miranda K., Bond D., McKee M., Skog J., Paunescu T., Silva N., Brown D., Russo L. (2010). Kidney Int..

[63] Hanke M., Hoefig K., Merz H., Feller A., Kausch I., Jocham D., Warnecke J., Sczakiel G. (2009). Urol. Oncology..

[64] Eissa S., Matboli M., Hegazy M., Kotb Y., Essawy N. (2015). Transl Res..

[65] Korzeniewski N., Tosev G., Pahernik S., Hadaschik B., Hohenfellner M., Duensing S. (2015). Urol. Oncol..

[66] Wang G., Chan E., Kwan B., Li P., Yip S., SzetoC. I.O., Ng C. (2012). Clin. Genitourin. Cancer..

[67] Dittmar M., Bischofs C., Matheis N., Poppe R., Kahaly G. (2009). J. Autoimmun..

[68] Eulitz D., Mannherz H. (2007). Apoptosis..

[69] Kishi K., Yasuda T., Ikehara Y., Sawazaki K., Sato W., Iida R. (1990). Am. J. Hum. Genet..

[70] Ito K., Minamiura N., Yamamoto T. (1984). J. Biochem..

[71] Nadano D., Yasuda T., Kishi K. (1993). Clin. Chem..

[72] Yasuda T., Awazu S., Sato W., Iida R., Tanaka Y., Kishi K. (1990). J. Biochem..

[73] Macanovic M., Lachmann P. (1997). Clin. Exp. Immunol..

[74] Mannherz H. (1992). J. Biol. Chem..

[75] Hitchock S. (1980). J. Biol. Chem..

[76] Calles-Escandon J., Cunningham J., Snyder P., Jacob R., Huszar G., Loke J., Felig P. (1984). Am. J. Physiol..

[77] Murai K., Yamanaka M., Akagi K., Anai M. (1980). J. Biochem..

[78] Yasuda T., Takeshita H., Nakazato E., Nakajima T., Hosomi O., Nakashima Y., Kishi K. (1998). Anal. Biochem..

[79] Ito K., Yamamoto T., Minamiura N. (1987). J. Biochem..

[80] Mizuta K., Yasuda T., Ikehara Y., Sato W., Kishi K. (1990). Z. Rechtsmed..

[81] Yasuda T., Sato W., Kishi K. (1988). Biochim. Biophys. Acta..

[82] Potenza N., Salvatore V., Migliozzi A., Martone V., Nobile V., Russo A. (2006). Nucleic Acids Research.

[83] Cranston J., Perini F., Crisp E., Hixson C. (1980). Biochim. Biophys. Acta..

[84] Rabin E., Weinberger V. (1975). Biochem. Med..

[85] Reddi K. (1977). Prep. Biochem..

[86] Spencer J., Schwaderer A., Dirosario J., McHugh K., McGillivary G., Justice S., Carpenter A., Baker P., Harder J., Hains D. (2011). Kidney Int..

[87] Iwama M., Kunihiro M., Ohgi K., Irie M. (1981). J. Biochem..

[88] Sakakibara R., Hashida K., Kitahara T., Ishiguro M. (1992). J. Biochem..

